# Valorisation of Whey and Buttermilk for Production of Functional Beverages – An Overview of Current Possibilities

**DOI:** 10.17113/ftb.57.04.19.6460

**Published:** 2019-12

**Authors:** Irena Barukčić, Katarina Lisak Jakopović, Rajka Božanić

**Affiliations:** University of Zagreb, Faculty of Food Technology and Biotechnology, Department of Food Engineering, Laboratory of Technology of Milk and Milk Products, Pierottijeva 6, 10000 Zagreb, Croatia

**Keywords:** whey, buttermilk, beverages, bioactive peptides, milk fat globule membrane (MFGM)

## Abstract

Whey and buttermilk are the main by-products of the dairy industry, both having excellent nutritional properties. Buttermilk contains a unique component, the milk fat globule membrane (MFGM). MFGM contains bioactive compounds with positive health effects like antitumour or cholesterol-lowering impact. Whey proteins are found in whey and are a source of bioactive peptides acting positively on coronary, gastrointestinal, immune and nervous systems. Yet, buttermilk and whey are insufficiently utilized in functional food production. Various technological solutions have been studied in order to increase the production of foods based on whey and/or buttermilk whereby the production of beverages appear to be most acceptable from the economic and technological point +of view. Thus, the aim of this paper is to give an overview of current knowledge about the possibilities of creating whey and/or buttermilk beverages.

## INTRODUCTION

Whey and buttermilk are the main by-products of the dairy industry. Despite their potential as raw materials for the development of new products, they are not used at all or very poorly. Whey originates from cheese production and contains highly valuable whey proteins, lactose, minerals and vitamin B (*1*, *2*). Whey proteins are rich in essential amino acids, which makes its biological value higher than of other proteins of animal and vegetable origin, including egg proteins, which were regarded as referent for a long period of time. Therapeutic value of whey has been known since the era of ancient Greeks and has been used for treatments of tuberculosis, hepatitis, diarrhoea, skin eczema, *etc*. (*3*, *4*). Buttermilk originates from butter production and contains lactose, proteins, milk fat globule membrane (MFGM), minerals and lecithin. Numerous studies have shown that MFGM contains bioactive compounds with antitumour and cholesterol-lowering effects, and inhibits *Helicobacter pylori* or prevents gastrointestinal infections. Buttermilk is usually processed by fermentation with lactic acid bacteria, while whey is placed on the market most commonly as pasteurised acid whey. Nevertheless, the quantities of buttermilk and whey available on the market are negligible. Both described by-products represent a very good basis for production of beverages of excellent nutritional and low energy value. Such properties are more than welcome in terms of modern consumer demands. Thus, the aim of this review is to highlight the necessity to increase the production of whey- and buttermilk-based beverages, as well to present current achievements in the research focused on whey and buttermilk processing into beverages.

## COMPOSITION AND CHARACTERISTICS OF WHEY AND BUTTERMILK

### Whey

Whey is a by-product of milk coagulation by acids and/or renneting enzymes during cheese or casein manufacturing. It is produced in volumes (80-90%) close to those of the processed milk used during cheese manufacture and therefore requires proper management (*1*). Depending on the processing technique used to separate casein from milk, there are two main types of whey, sweet whey with a pH=5.9–6.6 and acid whey with a pH=4.3–4.6. Whey has the total dry matter about 6-6.5%. It contains lactose (~70%), minerals (~12%), both depending on the acidity of the whey, whey proteins (~10%), vitamins and some fat (*2*). Whey also contains small quantities of components like organic acids, non-protein nitrogen compounds (urea and uric acid) and B group vitamins. Its composition and sensory characteristics mostly depend on the production process (acid or sweet), but also on the used milk (cow’s, sheep’s, goat’s, *etc*.), the season and the stage of lactation. The main differences are in the calcium, phosphate, lactic acid and lactate contents, which are higher in acid whey (*3*).

Whey has a very high polluting potential due to a high biochemical oxygen demand (BOD; 40-60 g/L) and high chemical oxygen demand (COD; 50-80 g/L), which are mostly conditioned by very high lactose contents in the dry matter (*4*-*6*). Thus, it has been estimated that the waste load of whey is 100-175 times higher than that of a similar volume of domestic waste water (*7*, *8*). Accordingly, adequate whey management is one of the priorities of the modern dairy processing sector.

Throughout the late 20th century a large body of scientific evidence indicated biological and technological value of whey. Such data were somewhat shocking to dairy processors, who treated whey as waste and disposed of it for decades. Nowadays, it is rather recognised as a highly valuable raw material that can be exploited by the agri-food, biotechnology, pharmaceutical and similar industries (*8*, *9*).

### Whey proteins – most valuable bioactive components of whey

Whey proteins are for sure the unique component responsible for the high nutritional and technological value of whey. They are mainly composed of thermosensitive fractions β-lactoglobulin (β-Lg), α-lactalbumin (α-La), blood serum albumin (BSA) and immunoglobulins. Thermostable proteose peptones as well as lactoferrin and lactoperoxidase are present in lower amounts too. Additionally, sweet whey contains caseinomacropeptide (CMP), resulting from chymosin (or pepsin) cleavage of k-casein. It constitutes approx. 20% of the sweet whey protein fraction and is not found in acid whey unless enzymatic coagulation was included in the process of manufacturing fresh cheese (*4*, *10*). Whey proteins are small globular proteins with amino acid profiles quite different from caseins due to high ratios of essential amino acids, smaller fraction of glutamic acid (Glu) and proline (Pro), but a greater fraction of sulfur-containing amino acid residues (*i.e*. cysteine and methionine) and higher contents of the branched chain amino acids (BCAA), which were recognized as important metabolic regulators in homeostasis of proteins, glucose and lipids (*7*, *8*). The parameters defining protein quality such as the protein digestibility-corrected amino acid score (PDCAAS) or biological value (BV) of whey protein exceed that of egg protein (the former benchmark), meat, fish, wheat and nuts (*9*). Accordingly, whey proteins have a superior nutritional value compared to common dietary sources of proteins.

Enzymatic hydrolysis of whey proteins in the human digestive system, fermentation of milk by starter cultures and hydrolysis by plant or microbial proteases result in the release of bioactive peptides. Once released, bioactive peptides act as signalling molecules and exert various physiological effects on the immune, gastrointestinal, cardiovascular and nervous systems (*11*, *12*). There is a growing body of scientific evidence confirming valuable influence of whey proteins on human well-being such as antimicrobial, antioxidant, antihypertensive, antidiabetic and immunomodulatory properties, and they may take part in mechanisms of reducing body mass and reduce or inhibit allergic reactions (*12*-*14*). The most abundant whey protein, β-Lg, is a source of multiple bioactive peptides like lactokinins possessing ACE-inhibitory ability, β-lactorphin acting as opioid agonist and ACE inhibitor; and β-lactotensin, which was proven to have an anti-stress effect, but also the binding ability to neuroreceptors responsible for satiety feeling regulation (*7*, *12*, *14*). α-La was demonstrated to be cytotoxic, to act protectively against mucosal injuries and oxidative stress, and have opioid, anti-inflammatory and anti-carcinogenic activity. Due to high tryptophane content, α-La can be used to improve sleep, mood and cognitive functions. More recently, a new genetic variant of α-La was isolated and identified, the so-called HAMLET (human alpha-La made lethal to tumour cells) that showed the ability to induce apoptosis of tumour cells while sparing healthy tissues at the same time (*7*, *15*). Among other whey protein fractions, lactoferrine should be mentioned as a source of lactoferricine – a bioactive peptide with confirmed antimicrobial, antiviral and immunomodulatory activity. Also, lactoperoxidase was demonstrated to possess high antimicrobial activity, while products containing this protein are successfully implemented in oral healthcare to promote healing of bleeding gums, gingivitis and oral irritation (*8*, *9*). In addition, whey proteins have excellent functional properties, such as good solubility, viscosity, gelling and emulsifying properties, and their concentrates are widely used in the food industry (*7*).

### Buttermilk

Buttermilk is the aqueous phase released during the churning of cream in butter production. In terms of ash and lactose content, buttermilk is similar to whey, but contains much higher amounts of proteins (*16*) (Table 1 (*3*, *17*)). However, they largely differ in terms of lipid fractions. Buttermilk contains about 4.6–14.5% fat in the dry matter (*18*), whereby a specific component derived from milk fat globule disruption during churning is present – the milk fat globule membrane (MFGM). Since MFGM is rich in phospholipids, buttermilk contains up to seven times higher concentrations (about 0.89 mg/g) of phospholipids than whole milk (about 0.12 mg/g) (*19*). Annual production of buttermilk in EU28 countries in 2016 accounted for 0.5 million tonnes (*20*).

**Table 1:** Comparative gross composition of sweet/acid whey and buttermilk (*3*, *17*)

*w*(constituent)/%	Sweet whey	Acid whey	Sweet cream buttermilk
Total solids Fat True protein Lactose Ash (minerals)	6.00 0.05 0.60 4.50 0.50	6.40 0.05 0.60 4.60 0.80	9.80 0.59 3.73 4.81 0.75

There are a few types of buttermilk that should be distinguished. Most of the studies have focused on investigating sweet buttermilk originating from churning of uncultured cream. There are also other types of buttermilk that can be produced such as sour buttermilk, obtained from churning of cultured cream, or whey buttermilk, originating from churning of whey cream during manufacture of whey butter. Whey buttermilk is produced in much smaller quantities than sweet buttermilk (*21*, *22*), but the potential market for whey buttermilk is huge considering the large volumes of whey produced annually.

Sodini *et al*. (*21*) determined the gross composition of sweet and sour cream buttermilk as well as of whey buttermilk. According to their results, sweet cream buttermilk had the highest content of total nitrogen (31.5%) and the lowest content of fat (13.1%). Whey buttermilk had the highest content of phospholipids and lactose. The lowest amount of nitrogen in whey buttermilk might be explained by the lack of casein in whey cream (*22*).

Libudzisz and Stepaniak (*19*) discussed the ability of buttermilk to increase the heat stability of recombined milk, mainly due to phospholipid-protein interactions preventing protein coagulation during sterilization. Thanks to casein and considerable amounts of phospholipids, buttermilk is characterised by good emulsifying properties suitable for wide use in the food industry (*22*, *23*).

In some countries like Russia, Poland, Czech Republic, Finland or Germany, naturally fermented buttermilk is merchandised as a beverage or is sometimes used for animal feed. Problems with increasing the consumption of traditional buttermilk is in the short shelf-life (about 1 week at 4–7 °C), in obtaining a uniform quality and in the lack of marketing promotion. Therefore, without putting a special effort to commercialize natural buttermilk, its consumption is destined to decrease (*21*).

### Bioactive components of buttermilk

During butter manufacture, milk fat globules are destabilized and disrupted by churning, which results in exclusion of the membrane covering the lipid matrix and its recovery in buttermilk along with other milk/cream components contained in the aqueous phase. Thus, a component known as MFGM is particularly rich in various proteins and phospholipids that possess an outstanding potential for functional and nutraceutical applications. Some of those are related to the prevention or amelioration of widespread chronic diseases such as cancer, obesity, diabetes and cardiovascular disorders (*24*, *25*). Buttermilk has received increased interest, as food science and nutrition research turn to develop products with not only increased nutritional value but also health-promoting properties. The MFGM has a complex composition and supramolecular structure (*26*). It is mostly constituted of protein fractions (average content about 1800 mg/100 g) followed by polar lipids (about 730 mg/100 g), although some other components like vitamin A, carotenoids, enzymes, adsorbed casein and whey proteins are present too (*27*). Major MFGM proteins are butyrophilin (BTN), xanthine oxidase/dehydrogenase (XO/XDH), mucin-like glycoproteins MUC1 and MUC15, adipophilin (ADPH), breast cancer type 1 and type 2 susceptibility proteins (BRCA1 and BRCA2). The most abundant polar lipids found in the MFGM isolated from buttermilk are phospholipids and sphingolipids, more exactly fractions of phosphatidylethanolamine, phosphatidylcholine, sphingomyelin, phosphatidylinositol and phosphatidylserine (*20*). Both of these have been proven to show numerous beneficial effects on the human health, some of which are presented in Table 2 (*20*, *27*-*29*).

**Table 2:** Overview of beneficiary effects of milk fat globule membrane (MFGM) proteins and polar lipids (*20*, *27*-*29*)

Component	Potential health effect
Fatty acid-binding protein	Cell growth inhibition Anticancer factor
Butyrophillin	Suppression of multiple sclerosis influences pathogenesis of autistic behaviour
Xanthine dehydrogenase/oxidase	Bactericidal agent Anti-inflammatory action
Breast cancer type 1 susceptibility protein	Inhibition of breast cancer
Breast cancer type 1 susceptibility protein	
Sphingolipids and metabolites	Shift in tumour type from malignant to benign Anticholesterolemic protection of the liver from fat- and cholesterol-induced steatosis Suppression of gastrointestinal pathogens (*Campylobacter jejuni*, *Listeria monocytogenes*, *Clostridium perfrigens*, *Escherichia coli*, *Salmonella enteritidis*) Neonatal gut maturation Myelination of the developing central nervous system Endogenous modulators of vascular function
Phosphatidylserine	Restoring normal memory of a variety of tasks Positive effects on Alzheimer patients (daily intake 200 mg) Improving exercise capacity of exercising humans
Phosphatidylcholine	Supporting liver recovery from toxic chemical attack or viral damage Protection of the human GI mucosa against toxic attack Reduction of necrotising enterocolitis
Mucins and glycoproteins	Inhibition of *Helicobacter pylori* Protective effect against rotavirus infections
MFGM hydrolysates	Antimicrobial activity against pathogens

Considering clinical trials, anticholesterolemic effect of sphingomyeline originating from MFGM has been examined since 1979, when Howard and Marks observed a significant decrease in serum cholesterol levels in patients who consumed cream in comparison to those who consumed butter (*29*). They concluded that such trends were most probably related to beneficial effects of MFGM, which is present in cream, but not detected in butter. Ito *et al*. (*30*) also conducted a clinical study and confirmed cholesterol-lowering effect in hypocholesterolemic mice fed with cream. Noh and Koo (*31*) found that hypercholesterolemic effect of sphingomyelin relies on its inhibitory action towards cholesterol absorption in the intestine. More recently, Baumgartner *et al*. (*32*) reported that a daily consumption of buttermilk during 12 weeks significantly lowered low density lipoprotein (LDL) cholesterol in individuals taking part in their clinical study. Conway *et al*. (*24*) reported that daily consumption of 45 g buttermilk significantly lowered LDL cholesterol levels in patients with hyperlipidemia. They also investigated the effects of buttermilk consumption on blood pressure and on the markers of the renin–angiotensin–aldosterone (RAS) system in humans (*25*). The obtained results were promising and confirmed that buttermilk consumption significantly reduced systolic blood pressure, the mean arterial blood pressure and plasma levels of the angiotensin I-converting enzyme compared to the placebo.

Thus, similarly to whey, buttermilk is also a nutritionally valuable coproduct with numerous potentially beneficial effects on human well-being. The production of buttermilk beverages should therefore be a key point when considering the future of milk-based beverages.

## PRODUCTION OF WHEY AND BUTTERMILK BEVERAGES

### Whey beverages

Production of whey-based beverages appears to be the most economical and simplest solution for whey utilization in human nutrition. The need for development of whey-based beverages is closely related to nutritional and functional properties of whey proteins, as well as to fulfilling the expectations of modern consumers who demand innovative products with enhanced functionality (*33*). Up to now, numerous research studies have focused on optimising whey beverage production from native or processed whey (*e.g*. powdered, deproteinated, diluted) (*34*). Along with technological development, the application of alternative food processing methods such as membrane processes, high intensity ultrasound or supercritical carbon dioxide technology is being investigated too. By applying nonthermal processing methods, hurdles like the low shelf life due to microbiological deterioration, occurrence of undesired sediments or whey protein denaturation might be overcome, but also the existing products could be improved (*9*, *35*-*37*). Over the past two decades, numerous whey-based beverages and similar products containing isolated whey components (mainly whey proteins) have been placed on the market as refreshing, value-added and/or functional foods (*33*).

### Nonfermented whey beverages

Although the easiest solution for designing a functional whey beverage seems to be using native sweet or acid whey as a basis, recently it has been suggested to use deproteinised whey or whey permeate remaining after ultrafiltration (*33*) in order to avoid undesirable sediment formation and blurring. Among the most frequently examined combinations is for sure the addition of orange juice to whey, often together with CO_2_. Chatterjee *et al*. (*38*) tested the production of a refreshing beverage consisting of concentrated whey and orange juice in various ratios. The mixture of orange juice and concentrated whey in ratio 3:2 appeared to be the optimal formulation with the best sensory properties. The shelf life at room temperature was determined to 11 days, while it lasted up to 3 months under cold storage conditions. Kumar and Bangaraiah (*39*) also tested the sensory properties, chemical composition and shelf life of multiple formulations of orange juice and whey. Thereby, two formulations (70% whey and 30% orange juice, and 65% whey and 35% orange juice) were evaluated with the highest sensory scores, while all of the produced beverages showed good microbiological stability and the potential to be stored for up to 2 months at room temperatures. Sady *et al*. (*40*) studied the production of whey beverage with orange juice and compared its properties to the same beverage produced without whey. The beverage containing whey was characterized by higher contents of proteins, ash, glucose, lactose and vitamin B, but contained less sucrose, fructose and vitamin C. Also, there were not any considerable differences in the polyphenolic content and the sensory score for colour desirability. Pareek *et al*. (*41*) investigated the production of refreshing carbonated beverage consisting of orange juice and whey in different ratios (70:30, 60:40 and 50:50). The mixture containing 70% orange juice and 30% whey was the most acceptable one and was characterised by a general increase in nutrients in comparison with the standard orange juice. The addition of tropical fruits or berries to whey has been investigated frequently. For instance, Jaworska *et al*. (*42*) added blackcurrant juice to acid whey and compared the characteristics between the pure blackcurrant juice and the one containing whey. The whey beverage with blackcurrant juice had higher amounts of ash, proteins and vitamin B_2_, while pure blackcurrant juice showed slightly higher antioxidant activity and received higher scores at sensory evaluation. Baccouche *et al*. (*43*) investigated the addition of prickly pear juice to acid whey. According to the obtained results, the beverages were physically stabilized by adding sugar and pectin to the heat-treated whey. Singh *et al*. (*44*) studied the production of whey beverage with guava consisting of approx. 68% whey and 20% guava pulp. The beverages were processed by pasteurisation at several temperature/time regimes and were stored in a cool place up to 90 days. The best beverage appeared to be the one pasteurized at 65 °C for 25 min and stored in a cool place for 45 days. Chavan *et al*. (*45*) optimised the production of a whey beverage with mango by mixing whey powder, whey protein concentrate or fresh whey with mango pulp or mango powder. The chemical composition, sensory properties and microbiological parameters of the produced beverages were analysed. The obtained results showed that regardless of prior processing (drying, concentrating), whey could be successfully utilized for beverage production, although a significant increase in acidity was detected in all samples. The beverage made from whey protein concentrate and mango powder showed good overall acceptability after 30 days of storage at refrigeration temperature. Valadão *et al*. (*46*) examined the possibilities of using ricotta cheese whey in sports drink production and found that tangerine, passion fruit and strawberry-passionfruit flavours achieved the best sensory scores. Janiaski *et al*. (*47*) optimised the production of fermented and nonfermented strawberry-flavoured whey beverages. The nonfermented strawberry-flavoured whey beverages were recognized as not enough acidic and viscous, having a more intense artificial strawberry aroma.

Some authors have studied the addition of herbs or spices in order to design a novel functional whey beverage. Yadav *et al*. (*48*) studied the production of whey beverage enriched with banana juice and *Mentha arvensis* extract. The optimal addition of *Mentha* extract was estimated at max. 2%, while the shelf life was determined to be 15 days. Similarly, Kumar *et al*. (*49*) developed a beverage from the ripe pineapple juice, whey and *Mentha arvensis* extract in the amount up to 3%. Refrigerated storability and BOD at room temperature of the produced beverages were analysed, while the possible changes were determined at 15-day intervals for up to 2 months. The most acceptable beverages were the ones containing between 0 and 1% *Mentha arvensis*, while its higher mass fraction caused a decrease in overall sensory quality. Satpute *et al*. (*50*) also incorporated *Mentha arvensis* extract into whey, but with beetroot pulp. There were four different types of beverages produced, and their chemical, sensory and microbiological parameters were analysed. Among all of the prepared beverages, the one consisting of 80% whey, 20% beetroot and 6% *Mentha arvensis* extract was evaluated as the best one. Alane *et al*. (*51*) optimised the preparation of a whey-based mango herbal beverage with the addition of ginger extract in volume fraction 0.5-5%. Thereby, the sample containing 10 g mango, 8 g sugar, 82 mL whey, 0.5% ginger extract and 0.05% guar gum achieved the highest overall acceptability. However, the microbiological quality of none of the produced beverage samples was satisfying during the tested storage period.

Regardless of the components chosen to create a refreshing whey beverage, the occurrence of sediment, salty sour taste and odour of cooked milk are still not avoided. Adding sufficient quantities of fruit base is critical for reaching the desired sensory quality, but on the other hand certain components of the fruit dry matter tend to precipitate and adversely influence beverage appearance. Thus, other solutions such as fermentation or non-thermal processing methods need to be reconsidered for application in whey beverage processing.

### Fermented whey beverages

Fermented beverages have been recognized by consumers all over the world for their therapeutic value. Considering the fact that whey contains almost 70% lactose from the milk, fermentation to yogurt-like drinks appears to be a meaningful way of whey utilization. Since fermentation process is accompanied by a decrease in pH due to transformation of lactose to lactic acid, sweet whey appears to be a better choice for fermented beverage production. Whey fermentation usually employs starter and/or probiotic cultures able to metabolise lactose such as *Lactobacillus acidophilus, Lactobacillus delbrueckii* ssp. *bulgaricus, Streptococcus thermophilus, Lactobacillus reuteri, Bifidobacterium bifidum, Lactobacillus rhamnosus, Propionibacterium freudenreichii* ssp. *shermanii, Lactobacillus casei, Lactobacillus plantarum, Lactobacillus helveticus, Enterococcus faecium, Bifidobacterium animalis* ssp. *lactis* and *Lactobacillus paracasei (**33**)*. Sohrabi *et al*. (*52*) studied the production of whey beverage fermented with a commercially available yoghurt culture DELVO^®^-YOG TY-17A DSL (DSM Food Specialties, Delft, The Netherlands). Thereby, whey protein concentrate was reconstituted and enriched by adding vitamin E prior to pasteurization and fermentation. At the same time, identical beverage was produced but without fermentation. Chemical, microbiological and sensory parameters of both drinks were analysed. Although there was no considerable difference in the chemical composition, fermented beverage achieved significantly higher overall acceptability and sensory scores. Very often probiotic whey beverages are investigated and are generally considered as one of the target segments of developing whey utilization for minimally processed functional products with added value (*53*). Among the most important factors is the chosen probiotic strain since it determines the unique flavour and texture of the fermented beverage. *Lactobacillus rhamnosus* is frequently used, but since it lacks the enzyme β-galactosidase, it does not have the ability to metabolize lactose. Therefore, it is often necessary to hydrolyse lactose prior to the fermentation or to use an appropriate co-culture. Pescuma *et al*. (*54*) studied the fermentation of whey protein concentrate by the strains *Lactobacillus acidophilus* CRL 636, *Lactobacillus delbrueckii* ssp. *bulgaricus* CRL 656 and *Streptococcus thermophilus* CRL 804, as single or mixed cultures. Fermented whey was then mixed with peach juice and calcium lactate and stored for 28 days at 10 °C. According to the obtained results, mixed cultures and single *Streptococcus thermophilus* CRL 804 culture showed a good surviving potential during the tested storage period. Also, all of the tested strains degraded β-lactoglobulin (41–85% after 12-hour incubation), which is of a great importance since β-Lg B is one of the major milk allergens. Koç *et al*. (*55*) fermented whey with probiotic strains *Lactobacillus plantarum* or *Lactobacillus brevis* and their combination. Fermented whey was supplemented with different fruit concentrates (lemon, mango, pineapple, apple or grape) and sucrose in order to mask the bitter flavour and achieve acceptable sensory characteristics. According to the obtained results, a beverage inoculated with *Lb. plantarum* and enriched with pineapple concentrate was the most preferred one. Seyhan *et al*. (*56*) studied the production of fermented whey beverages from whey powder fortified with soy isoflavones or phytosterols and inoculated with probiotic strains *Lactobacillus acidophillus* La-5 or *Lactobacillus casei* LBC-81. The addition of nutraceuticals did not change the basic composition of the produced beverages, but the phytosterol-fortified beverages were significantly more acceptable in terms of sensory quality and would be suitable for industrial-scale production. Similarly, Schlabitz *et al*. (*57*) studied the shelf life of fermented probiotic products produced from different mixtures of whey and powdered milk. The beverages were produced by inoculation with probiotic strains *Lactobacillus acidophilus* LA-5, *Bifidobacterium animalis* ssp. *lactis* BB-12 and *Streptococcus thermophilus.* They were fortified by adding prebiotics, strawberry pulp and strawberry flavour. Eleven formulations were developed and their chemical, microbiological and sensory parameters were analysed. The obtained results confirmed the possibility of producing a fermented probiotic beverage containing up to 70% ricotta cheese whey. Yasmin *et al*. (*58*) found that fermented whey beverages supplemented with probiotics play an important role in lowering cholesterol levels in rats. Faisal *et al*. (*59*) investigated the production of fermented probiotic whey beverages enriched with orange powder. Beverages were produced using the fresh cheddar cheese whey supplemented with orange juice powder and orange flavour and fermented at 42 °C by a combined thermophillic probiotic culture consisting of strains *Lactobacillus acidophillus* La-5, *Lactobacillus delbrueckii* ssp. *bulgaricus*, *Streptococcus thermophilus* and *Bifidobacterium* sp. BB-12. The best ranked beverage consisted of 1 L cheese whey, 0.70 g stabilizer, 8% sugar, 1% orange powder and 0.40 mL ﬂavour. The authors concluded that the addition of orange ﬂavour and sugar into whey fermented by probiotic strains might be a successful method for utilizing cheddar cheese whey for beverages with acceptable sensory characteristics. Skryplonek and Jasińska (*60*) and Skryplonek (*61*) studied the potential of acid whey in fermented drink production. Acid whey was inoculated with yoghurt culture and probiotic strains *Lactobacillus acidophilus* La-5 and *Bifidobacterium animalis* ssp. *lactis* BB-12. For further supplementation, buttermilk powder, sweet whey powder, condensed milk, UHT milk and skimmed milk powder were tested. The obtained results showed that acid whey could also be used as a raw material to manufacture fermented probiotic beverages and also provide sufficient levels of bacteria required to ensure health benefits to consumers.

Recently, some studies have focused on production of kefir-like whey beverages. Pereira *et al*. (*62*) studied the fermentation of ultrafiltered whey protein concentrates by kefir grains and/or selected probiotic strains. The fermented drinks were acceptable according to their physicochemical and sensorial properties, and contained satisfactory counts of microorganisms after 14 days of refrigerated storage. Magalhães *et al*. (*63*) compared kefir produced from milk with the one from whey by using kefir grains. Lactose hydrolysis, ethanol levels, formation of organic acids and volatile compounds determined during fermentation were compared among samples of milk kefir and whey kefir. The obtained results indicated that kefir grains utilized lactose from whey and deproteinised whey and produced similar amounts of ethanol, lactic acid and acetic acid to those obtained during milk fermentation. Thus, whey could be a valuable substrate for production of kefir-like beverages.

### Application of non-thermal processing methods in whey beverage production

In recent years, lots of efforts have been put into optimizing the application of non-thermal processing methods into whey processing. In order to partially or entirely prevent the undesired advents such as sediment formation, poor mouthfeel, salty sour taste, *etc*., different attempts have been made including for example the application of membrane processes or whey treatment by high intensity ultrasound. Barukčić *et al*. (*36*, *64*) examined the possibility of applying microfiltration and ultrafiltration in order to achieve adequate microbiological stability without whey protein denaturation and sediment formation. Promising results were obtained by combining microfiltration with a 0.5-µm membrane and a subsequent ultrafiltration with a 0.2-kDa membrane. Both membranes were ceramic and the process was performed at 20 °C. Optimal microbiological quality was obtained and the nutritional quality was almost entirely preserved, meaning that whey proteins were maintained in the native state. Such findings were better than those achieved by the usually applied pasteurization at 73 °C for 20 s, which opened new possibilities for producing minimally processed whey-based beverage of excellent nutritional quality. Ultrafiltration or reverse osmosis allow whey concentration and could solve the problem of the poor mouthfeel inherent to whey beverages (*65*). Accordingly, Dilipkumar and Yashi (*66*) investigated a production of refreshing fruit beverage using the native, prefiltered and ultrafiltered (UF) acid whey as basis. Thereby, the addition of mango, pineapple and orange juice in different amounts (18, 20, 22 and 24%) was tested. The best properties and overall acceptability among consumers were recorded for the carbonated beverage consisting of UF whey basis enriched with the addition of 22% pineapple juice. Since the implementation of membrane processes eludes great nominal financial expenses, they are mainly used for the production of more cost-effective products such as whey protein concentrates or isolates. In order to establish a production without waste products, many authors have proposed the utilization of the remaining UF permeate. Beucler *et al.* (*67*) investigated the application of whey permeate for the production of carbonated beverage enriched with addition of different flavours and vitamins. Amaral *et al*. (*37*) investigated the application of supercritical carbon dioxide for application as alternative technology to thermal processing during production of whey and grape juice. They found no differences in the characteristics (acidity, total solids, antioxidant activity) of the juice processed by conventional heat treatment and supercritical carbon dioxide technology. High intensity ultrasound was also investigated for purposes of preventing sediment formation or reducing its amount, for improving the fermentation or for partial substitution of pasteurization (*35*, *68*). Barukčić *et al*. (*68*) investigated the influence of high intensity ultrasound on the quality and fermentation of reconstituted sweet whey. In the ﬁrst stage of the study, whey was subjected to treatments with different power inputs (480 and 600 W) for 6.5, 8 and 10 min at constant temperature (45 and 55 °C). Microbiological quality, particle size distribution, protein content, acidity, electrical conductivity, viscosity and sensory properties of the treated whey samples were analysed. All of the parameters were compared to the control sample (pasteurized) and to fresh whey. Whey thermo-sonication by nominal power of 480 W for 10 min at 55 °C resulted in better microbiological quality and sensory properties than whey pasteurization. Inﬂuence of high intensity ultrasound on whey fermentation with yoghurt culture YC-380 and with monoculture *Lactobacillus acidophilus* La-5 (both Christian Hansen, Hørsholm, Denmark) was investigated. Different ultrasound treatments were applied for culture activation prior to or after the inoculation, and treatment with nominal input power of 84 W for 150 s resulted in the highest increase of the viable count during the activation process. Jeličić *et al*. (*35*) studied the application of a combination of high intensity ultrasound with nominal inlet power of 400 W with moderate heat (55 °C) in whey processing. The applied treatment resulted in a better reduction of the number of total bacteria, coliform bacteria, yeasts and moulds than conventional pasteurization. Also, sensory properties were improved regarding mouth feel, absence of sediment and unchanged colour in all of the ultrasound-treated whey samples.

Thus, along with the development of new processing technologies, whey utilization could be improved too. There are numerous scientific studies that have proven the feasibility as well as the importance of producing whey-based beverages and similar products, not only for resolving the question of environmental pollution, but also for its outstanding nutritional value.

### Buttermilk beverages

#### Nonfermented buttermilk beverages

According to its composition, buttermilk has a great potential for utilization in producing innovative food products based on milk protein and carbohydrate. Knowing these facts, Meshram (*69*) studied the production of buttermilk-based fruit beverages containing different amounts of mango, orange and banana. The composition of an acceptable beverage formulation mostly depended on the type and the amount of fruit juice, which ranged from 10% for mango to 35% for orange, while the buttermilk content of the beverages ranged from 62 to 83%. Among all tested beverages, mango buttermilk beverage was found to be most acceptable. It contained 12% mango juice, 81% buttermilk, 7% sugar, 0.05% pectin and 0.12% carboxymethyl cellulose (CMC), with pH=4.25. Buttermilk can also be very well utilised in milkshakes, since it contains considerable amounts of phospholipids which decrease surface tension on ’liquid-air’ boundary and assist foamy structure creation at mechanical beating of buttermilk (*70*). Buddhadasa *et al*. (*71*) investigated the addition of fruit juices or pulp in the production of buttermilk-based beverages. Soursop (*Annona muricata*) is a fruit rich in vitamin B, potassium, fructose and vitamin C, and is also proven to have anticancerogenic properties as well as broad spectra of antimicrobial activities against bacterial and fungal infections, and is used to treat depression and stress. According to the obtained results, the addition of 13% soursop and 12% sugar was recognized as optimal for the production of a functional buttermilk beverage. Mudgil and Barak (*72*) optimized buttermilk production enriched with 4% soluble fibre (partially hydrolysed guar gum) and thus improved its physicochemical and sensory characteristics. Fibre fortification of buttermilk considerably reduced the phase separation, which is a serious problem in reference to consumer acceptance. Kiruthika *et al*. (*73*) investigated the production of buttermilk-based beverage supplemented with pearl millet paste, Bengal gram flour and spices (cumin, salt, ginger extract, asafoetida, green chilli extract, curry leaves and coriander leaves). The physicochemical, microbiological and sensory properties of all beverages were tested during storage for 7 days. The optimal ratio of buttermilk to paste was determined at 80:20, while the produced beverage was described as superior compared to sole buttermilk due to better sensory properties and richer nutritional composition.

Similar to whey, some efforts have also been made in developing carbonated beverages. Shaikh and Rathi (*74*) investigated the development of fruit-flavoured carbonated buttermilk beverages. According to the obtained results, sensory, physicochemical and nutritional parameters were improved by the addition of mango, pineapple and orange fresh juices. The best combination, evaluated by the sensory panel, was the fruit-flavoured carbonated buttermilk beverage prepared with the addition of 12% sugar and 24% pineapple juice.

#### Fermented buttermilk beverages

Cultured buttermilk is a by-product of butter production from cultured cream, but can also be produced by fermentation of sweet buttermilk. Sweet buttermilk used for buttermilk production should be of the highest quality from the compositional and microbiological points of view.

Starter cultures for the production of fermented buttermilk usually contain strains of *Lactococcus lactis* ssp. *lactis, Lc. lactis* ssp. *cremoris* and *Leuconostoc mesenteroides* ssp. *cremoris* (*75*). These species produce mainly lactic acid and are often referred to as acid producers, in contrast to *Leuconostoc* spp., which ferment citric acid and produce important metabolites, such as CO_2_, acetaldehyde and diacetyl, which are referred to as aroma and flavour compounds (*22*). It is most important to achieve the balance between the aroma and intensity. Wróblewska *et al*. (*75*) reported that max. 20% of aroma-producing bacteria are recommended for use to achieve diacetyl content of 2-5 mg/kg, which is responsible for the characteristic ‘buttery’ flavour and aroma. Therefore, supplementation of milk with citrate or citric acid is often recommended. Another very important flavour compound from the carbonyl group produced by lactic acid bacteria in buttermilk is acetaldehyde, which is undesirable and if present in excess, responsible for the flavour defect described as green or yogurt-like. In starters for the production of buttermilk, it is better to use *Leuconostoc mesenteroides* ssp. *cremoris* rather than *Lactococcus lactis* ssp. *lactis* for flavour production. Generally, in order to obtain buttermilk with desirable organoleptic properties, the optimum ratio of diacetyl and acetaldehyde needs to be around 4:1 (*18*). The important sensory characteristics of buttermilk resulting from mesophilic fermentations are the typical consistency, which is due to the coagulation of milk proteins by lactic acid, and the aroma and flavour produced by the fermentation of citric acid and lactose. Fermented buttermilk is characterized by thick, smooth, fairly viscous properties. The texture also depends on whether polysaccharide-producing strains are included in the starter culture and on the concentration of total solids (*18*).

De Bassi *et al*. (*76*) evaluated three fermented milk beverages supplemented with sugar and strawberry puree after the fermentation. The base contained 70% milk, with whey and buttermilk added in combinations 30 and 0%, 15 and 15%, and 0 and 30%, respectively. The chosen starter culture developed well in all beverage formulations reaching pH=4.7-4.9 within 180 min of fermentation. Lactic acid bacteria in the products were above 8 log CFU/mL throughout the storage period, while there were no considerable differences in the pH, acidity and viscosity. The authors concluded that buttermilk and whey could be interesting ingredients for fermented beverage production, because the consumers liked all the products equally. Mudgil *et al*. (*77*) studied the production of cultured buttermilk beverage supplemented with *Aloe vera* juice. Viscosity, phase separation, acidity, pH and sensory properties of the produced beverage was analyzed. There were no significant differences in acidity, but it was noticed that *Aloe vera* juice decreased the phase separation and increased the viscosity of the beverage. Samples with 10% *Aloe vera* juice obtained the highest scores in the sensory evaluation and had improved nutritive and physicochemical properties.

Sheth and Hirdyani (*78*) discussed the development of a buttermilk-based fermented drink fortified with barley and fructooligosaccharides (FOS) as functional ingredients. Barley was pre-cooked, added to buttermilk and fermented, after which FOS, different flavours (rose, khus, chocolate and salt-jeera) and colours were added. A panel of 24 trained members evaluated the colour, appearance, mouthfeel, texture, taste, aftertaste, and overall acceptability of the beverages. According to the obtained results, there were no significant differences among the drinks with different flavours, but salt-jeera flavour was the most appreciated by all panel members, followed by rose. No aftertaste or bad mouthfeel was reported in any of the beverages.

## WHEY AND BUTTERMILK BEVERAGES AS HEALTH PROMOTING FOODS

Taking into consideration the constantly increasing number of data published about investigating properties of whey and buttermilk as well as options for their utilization, it is obvious that both of these by-products have more than a promising potential for functional food production. Considering the production costs and the effects of processing methods such as drying at high temperatures on nutritional properties, production of beverages seems to be the logical solution for utilization of whey and buttermilk, especially since it has been extensively researched over the past ten years (Table 3 (*39*-*41*, *44*, *48*, *49*, *55*, *74*, *77*, *79*, *80*)). In addition, functional beverages are popular among various consumer groups either as meal replacements or for purposes like rehydration after exercise, refreshment and rehydration during exposure to high environmental temperatures, easily available and ready-to-eat healthy snacks, *etc*. These are mostly consumed by athletes, health-conscious working population and consumers eager to include healthy alternatives into the everyday diet (*81*). Compared to non-diary beverages, whey and buttermilk are considered as a pool of bioactive proteins, peptides and/or lipids with numerous health-promoting activities such as antimicrobial, anticancerogenic, antihypertensive, antiulcerative, antitumour, antidiabetic, cholesterol-lowering and immunomodulatory. Besides, they are also rich in B-group vitamins, minerals, especially calcium, sodium and potassium, and lactose – a disaccharide with relatively low glycemic index (*26*). Therapeutic value of whey has been known for centuries and has already been exploited for healing diarrhoea, biliary diseases, arthritis, various intoxications, anaemia, liver and skin diseases in specialized institutions that were intensively established in Switzerland, Germany and Austria during the 18th century (*82*).

**Table 3:** List of some recently reported studies on development of beverages based on whey or buttermilk

Beverage formulation	Outcomes	Reference
Orange juice and whey in different ratios	Best sensory and microbiological properties for formulations (in %)70 whey/30 orange juice and 65 whey/35 orange juice	(*39*)
	Whey orange juice characterised by higher content of proteins, ash and vitamin B than orange juice	(*40*)
	Beverage containing 70% whey, 30% orange juice and CO_2_ achieved best sensory scores and had the best nutritional composition	(*41*)
Different formulations of guava pulp and whey	The best beverage was obtained by pasteurisation at 65 °C for 25 min and cold storage for 45 days	(*44*)
*Mentha arvensis* extract addition to whey	Optimal beverage contained banana juice and max. 2% *Mentha arvensis* extract, shelf life of 15 days	(*48*)
	Best formulation consisted of 80% whey, 20% beetroot and 6% *Mentha arvensis* extract	(*49*)
Whey fermentation by probiotic strains *Lactobacillus plantarum*, *Lactobacillus brevis* and their combination and supplementation with fruit concentrates (lemon, mango, pineapple, apple, grape) and sucrose	Optimal beverage inoculated with *Lb. plantarum* and enriched with pineapple concentrate	(*55*)
Five mixes of probiotic drinks formulated from sweet whey and black mulberry (BM) juice	Viability of probiotic strains *Lactobacillus rhamnosus* GG and *Biﬁdobacterium animalis* ssp. *lactis* Bb-12 remained high up to 14 days of cold storage; sensory properties of sample with 25% whey and 75% BM juice obtained the highest scores	(*79*)
Addition of CO_2,_ and fresh mango, pineapple and orange juices to buttermilk	Best formulation was obtained by adding 12% sugar and 24% pineapple juice to buttermilk	(*74*)
Cultured buttermilk beverage supplemented with *Aloe vera* juice	Samples containing 10% *Aloe vera* juice obtained the highest sensory scores and had improved nutritive and physicochemical properties compared to sole buttermilk	(*77*)
Therapeutic buttermilk formulation by fermentation and incorporation of *Moringa* pod powder (MPP)	Buttermilk fermented with mesophilic/thermophilic dahi culture, supplemented with 1.92% MPP was found optimal due to satisfying shelf life of 20 days at refrigeration and high content of calcium, iron and vitamin A	(*80*)

Nowadays, there is an increasing occurrence of diseases related to stress, unhealthy diet and/or busy lifestyle such as atherosclerosis, obesity, diabetes, osteoporosis, *etc*. Knowing the aforementioned properties, buttermilk- and whey-based beverages might be an excellent choice for overcoming these disorders. Some clinical trials have already demonstrated their effectiveness in reducing and controlling blood pressure and serum cholesterol levels (*24*, *25*, *32*, *83*), regulation of blood sugar levels in diabetes type 2 patients (*84*), reduction and control of body mass (*85*, *86*), supporting recovery of children suffering from malnutrition (*87*), *etc*. Accordingly, whey and buttermilk are much more than byproducts of dairy industry and their valorisation into functional beverages with exceptional nutritional value should be one of the target points in creating the future of dairy products.

## CONCLUSION

Whey- and buttermilk-based beverages target a large scale of consumers - from little children to old people. Their nutritional properties and potential health-promoting effects meet the requirements of modern consumers who are increasingly becoming aware of the great influence food has on humans. Beverages containing a certain amount of fruits or carbonated beverages have gained the most attention up to now, and were evaluated as acceptable to the consumer. Besides, very large interest has also been noted for fermented beverages, especially when containing probiotic strains. Thus, there are many options for creating whey- or buttermilk-based beverages that are appealing to the consumer. However, the main problem seems to be in the lack of information available to the consumers considering the advantages of these products. In turn, there is insufficient interest for such beverages, which makes the reviewing of their production rather risky from the dairy industry point of view. So, future perspective lies in putting more efforts into marketing and informing activities in order to draw the consumer attention to the outstanding nutritional and functional properties of whey and buttermilk.
